# Associations between job demand-control-support and high burnout risk among physicians in Sweden: a cross-sectional study

**DOI:** 10.1186/s12995-024-00441-6

**Published:** 2024-10-29

**Authors:** Filip Christiansen, Britta Elsert Gynning, Abid Lashari, Josefina Peláez Zuberbühler, Gun Johansson, Emma Brulin

**Affiliations:** ¹Institute of Environmental Medicine, Karolinska Institute, Stockholm, Sweden

**Keywords:** Occupational stress, Burnout, Physicians, Healthcare

## Abstract

**Background:**

The knowledge about job demands, control, and support, and their potential associations with burnout risk among physicians in Sweden, is limited. This study aimed to explore (i) factors of the JobDemand-Control-Support (J-DCS) model across different groups of physicians in Sweden, (ii) their association with high burnout risk, and (iii) the potential buffering impact of job control and support.

**Methods:**

Cross-sectional data from the Swedish Longitudinal Occupational Health in Healthcare Survey (LOHHCS) study cohort was used. In 2021, a total of 2032 respondents submitted questionnaire data comprising J-DCS measures (i.e., job demands, workplace control and task-level control, and social support from peers and managers). Burnout risk was measured using the Burnout Assessment Tool. Binary logistic regression models were used to investigate the associations between the J-DCS variables and high burnout risk. Interaction analysis was performed to explore any moderation of the associations.

**Results:**

Job demands were significantly associated with increased odds of high burnout risk (odds ratio (OR) 2.71, 95% confidence interval (CI) 1.91–3.84. Workplace control (OR 0.50, 95% CI 0.35–0.71) and peer support (OR 0.61, 95% CI 0.48–0.77) were significantly associated with reduced odds of high burnout risk. The interaction analysis showed no significant moderation of the association between job demands and high burnout risk by either peer support or workplace control, and no buffering impact was found.

**Conclusion:**

Job demands were associated with high burnout risk among physicians in Sweden. Although workplace control and peer support had inverse associations with high burnout risk, no moderation or buffering impact on the association between job demands and high burnout risk was found. Longitudinal studies are needed to confirm these associations.

**Supplementary Information:**

The online version contains supplementary material available at 10.1186/s12995-024-00441-6.

## Background

Globally and in Sweden, physicians are exposed to high levels of work-related stress, resulting in a high prevalence of burnout [1–3]. Burnout is a state caused by a prolonged negative psychological response to workplace stressors, and includes symptoms of exhaustion, mental distance, emotional and cognitive impairment [4]. In Sweden, up to 28% of physicians experience exhaustion [5], an average of 14% are at risk of burnout [3], and 4.7–6.9% are at high risk of burnout [5, 6]. This adverse situation may have several negative consequences for physicians, patients, and the healthcare system in general [1]. Increased knowledge of work-related factors associated with high burnout risk among physicians in Sweden is required to ultimately find ways of mitigating its negative effects.

Several psychosocial factors at work may contribute to burnout risk. For example, among physicians in Sweden, an imbalance in job efforts and rewards, and work-life interference, is associated with increased burnout risk [3, 7]. Over the past decades, the working conditions for Swedish physicians have deteriorated, characterized by an increase in the number and altered nature of work tasks, less influence in decision-making, and reduced support [8]. The well-established Job Demand-Control-Support (J-DCS) model posits that high job demands (e.g., stress-inducing factors, work task frequency and intensity) and low job control (e.g., decision-making autonomy, skill discretion) are linked to an elevated risk of negative health outcomes, including burnout [9]. Specifically, for physicians, excessive workloads combined with low job control appear to be key contributors to burnout [10]. However, several previous studies indicate that social support may mitigate the negative effects of work-stress exposure [11–14]. Notably, approximately 33% of physicians in Sweden experience low social support from managers, and 14% experience low peer support [15].

The J-DCS has been studied in relation to physician burnout in other countries [16], yet this association remains unexplored among physicians in Sweden [3, 5, 6]. Since healthcare systems differ significantly in financing, care delivery, and organizational structures [17], the distribution of work-related stressors may vary largely across countries, highlighting the importance of further exploration of J-DCS among physicians in Sweden. Recent evidence suggests that J-DCS varies between occupational groups within Swedish healthcare. For example, physicians experience higher quantitative job demands compared to nurses, yet they report greater work-time control and influence [18]. Conversely, physicians experience less peer support but higher levels of managerial support [18]. While levels of burnout are known to differ among various physician subgroups [3, 5], detailed analyses of how J-DCS factors are distributed across these groups are still lacking. Meanwhile, recent findings challenge the theoretical framework of the J-DCS model by suggesting that the protective effects of job control and support may diminish under high job demands [19], although these potential mechanisms have not been investigated among physicians in Sweden.

Against the backdrop of previous studies both supporting and questioning the J-DCS model [10–13, 19], the present study will test this model in relation to high burnout risk among physicians. Specifically, the present study aimed to explore (i) psychosocial factors of the J-DCS model across different groups of physicians in Sweden, (ii) their association with high burnout risk, and (iii) the potential buffering impact of job control and support. Addressing this knowledge gap is important to inform policymakers and healthcare leaders in designing strategies to promote healthier work environments and implement effective preventive measures against work-related stress in Swedish healthcare.

## Methods

### Study design, participants and data collection

**Fig. 1:**
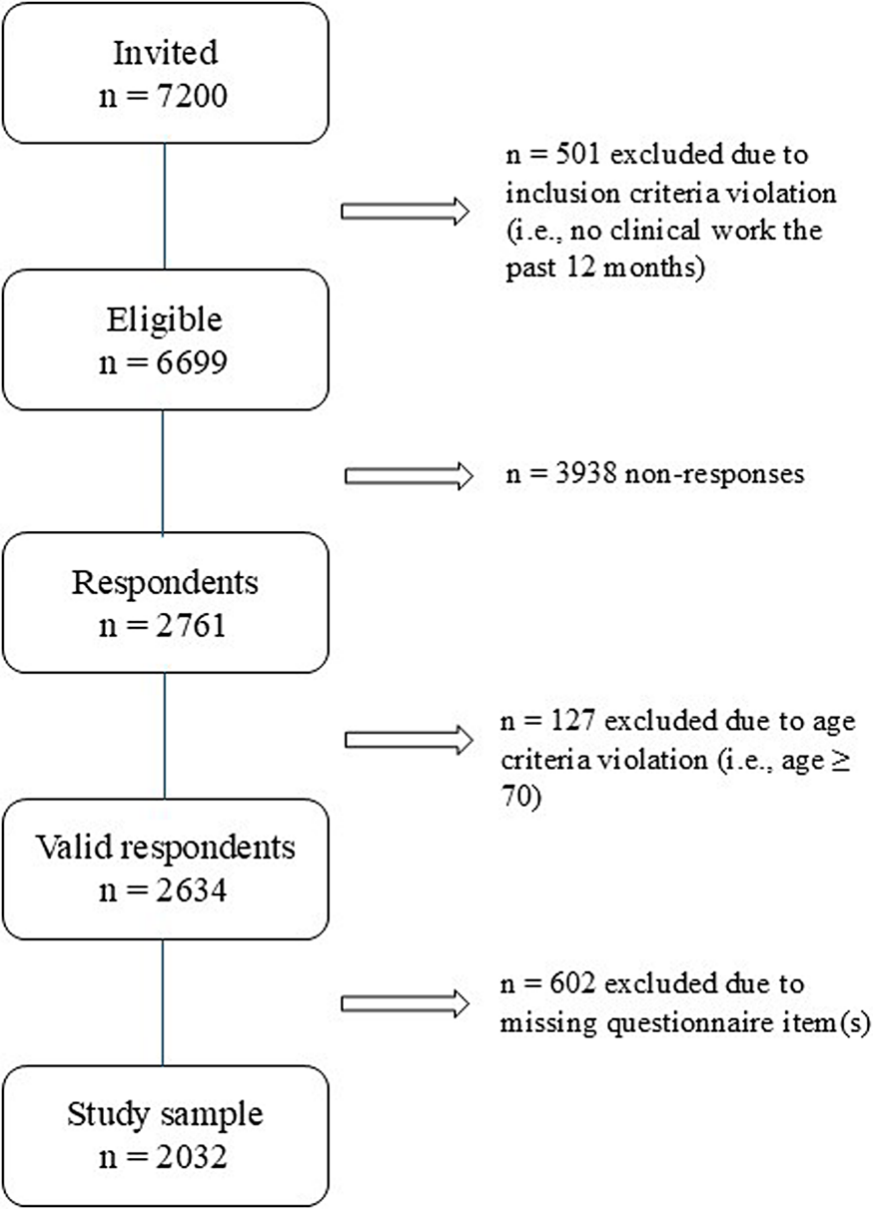
Flow chart

This study was based on cross-sectional data from the Swedish Longitudinal Occupational Health in Healthcare Survey (LOHHCS) [5]. The LOHHCS cohort consists of a representative sample of physicians in Sweden based on the Swedish Occupational Register (as of 2018) [20], managed by the Swedish government agency Statistics Sweden [20]. In 2021, a total of 7200 physicians were invited based on a stratified random sampling from 12 strata, comprising six administrative healthcare regions and two work site conditions (i.e., primary care facility or hospital) in Sweden. In total, 501 participants were originally excluded from the LOHHCS study due to inclusion criteria violation (i.e., no active clinical duty during the past 12 months), resulting in 6699 participants. During February and May 2021, Statistics Sweden collected and processed questionnaire data from 2761 valid respondents (response rate = 41.2%). For details regarding the LOHHCS study population and data collection, please see a previous publication by Hagqvist et al. [5]. In this study, the analytical sample was further restricted by age criteria (i.e., age < 70), removing 127 participants. Additionally, questionnaire data submission failure or missing values in exposure or outcome variables resulted in the exclusion of 602 participants (Fig. 1). In total, the sample of the present study consisted of 2032 participants.

The present study was approved by the Swedish Ethical Review Authority (2020–06613).

### Measurements

#### Job demand-control-support

Job demands and support were assessed using the core items from the validated Copenhagen Psychosocial Questionnaire III (COPSOQ III) [21], scored on a Likert basis from 1 to 5 (i.e., 1 = Always/to a very large extent, 5 = Never/to a very small extent). The job demands and support items were reversibly coded to reflect high job demands and high support for high scores (i.e., Always/to a very large extent = 5, Never/to a very small extent = 1). In total, job demands were measured with four items (addressing the distribution of workload, time to complete work tasks, work pace, and quantity of work tasks, respectively). Cronbach’s alpha (α) was 0.816 for job demands, indicating high internal consistency. Social support was measured with one item for support from peers, and one item for support from managers, respectively.

To capture healthcare-specific job control, two core items from the COPSOQ III [21] were combined with one validated item regarding professional autonomy [22], and three additional questionnaire items addressing physician’s ability to impact their work. These additional three items were added by the research group for contextual purposes, based on methodological reasoning of the job control aspect for physicians in line with the general J-DCS conceptualization [9]. In total, the following six items addressed job control:

COPSOQ III items [21].

i) Workplace information regarding decisions, changes, and plans.
ii) Workplace facilitation of efficient work.

Professional autonomy item [22].

iii) Workplace facilitation of clinical decision-making.

Additional questionnaire items addressing physician’s ability to:

iv) Impact the number of patient consultations per day.
v) Impact the time frame of each consultation.
vi) Impact the time for administrative work and documentation.

All job control items were answered on a Likert basis from 1 to 5 (i.e., 1 = Always/to a very large extent, 5 = Never/to a very small extent) and reversibly coded (i.e., high scores reflected high job control). Exploratory factor analysis (EFA) was conducted on all items according to guidelines (i.e., retaining factor loadings ≥ 0.40) [23]. The EFA rendered two factors, each including three items of the job control aspect, defined as workplace control (comprising items i-iii above) and task-level control (comprising items iv-vi above). Cronbach’s α was 0.709 and 0.869 for workplace control and task-level control, respectively, indicating sufficient internal consistency for both job control variables. Please see Supplementary Material for a detailed overview of the EFA output, including factor loadings.

#### Burnout

The Burnout Assessment Tool (BAT), defined by Schaufeli et al. [24], was used to measure burnout risk. The BAT contains 23 items across four categories of symptoms of burnout (i.e., exhaustion, mental distance, emotional impairment, and cognitive impairment) [24]. The BAT provides high reliability and validity and has been psychometrically tested in several countries in close proximity to Sweden [25]. In addition, it provides a cut-off value for high burnout risk based on a score across all symptoms of burnout [26]. All 23 items of BAT were answered on a Likert basis (i.e., 1 = No, never, 5 = Yes, most of the time). Cronbach’s α for all 23 items of BAT was 0.948, indicating high internal consistency. binary outcome variable representing high burnout risk was created i.e., coded as 1 for mean BAT-score ≥ 3.02, indicating a high burnout risk, and 0 for no burnout risk, according to recent recommendations by Schaufeli et al. [27].

#### Sociodemographic and occupational variables

Sociodemographic variables comprised sex (men and women), age (divided into quartiles and as a continuous measure) and having a partner and/or kids (yes or no). Occupational characteristics comprised the variables sector (public- or private sector), rank (physicians in training (i.e., junior-, intern- and resident physicians or equivalent), specialist physicians and consultants, respectively), worksite (primary care facility, hospital, or other), years of clinical work experience (< 5, 5–10, 11–15, > 15), average working hours per week (< 30, 30–40, 41–50, > 50), and exposure to regular shift work (yes or no). Significant group differences are shown in Table 1.

**Table 1:** Descriptive statistics and scores of Job demand-control-support and the Burnout Assessment Tool Across Sociodemographic and Occupational Characteristics of Physicians in Sweden

	Total	Demands	Workplace Control	Task-level Control	Peer Support	Manager Support	BAT score
	*n (%)*	*mean (SD)*	*mean (SD)*	*mean (SD)*	*mean (SD)*	*mean (SD)*	*mean (SD)*
**Sex**							
Male	893 (44)	3.39 (0.80)	3.77 (0.69)	2.53 (1.09)	4.33 (0.81)	3.85 (1.10)	1.83 (0.60)
Female	1139 (56)	3.63 (0.74)	3.62 (0.67)	2.33 (1.08)	4.25 (0.83)	3.67 (1.12)	2.01 (0.61)
**Age**							
27–37	551 (27)	3.49 (0.76)	3.54 (0.71)	2.28 (1.03)	4.43 (0.75)	3.70 (1.12)	2.00 (0.65)
38–45	507 (25)	3.60 (0.71)	3.65 (0.63)	2.30 (1.07)	4.33 (0.75)	3.80 (1.05)	1.95 (0.58)
46–57	509 (25)	3.64 (0.75)	3.75 (0.67)	2.39 (1.07)	4.21 (0.86)	3.69 (1.13)	1.98 (0.63)
58–69	465 (23)	3.36 (0.86)	3.84 (0.70)	2.76 (1.13)	4.14 (0.89)	3.80 (1.17)	1.77 (0.54)
**Partner**							
Yes	1844 (91)	3.53 (0.77)	3.69 (0.68)	2.44 (1.09)	4.30 (0.80)	3.76 (1.10)	1.92 (0.60)
No	188 (9)	3.53 (0.80)	3.66 (0.73)	2.23 (1.03)	4.12 (0.97)	3.66 (1.21)	2.05 (0.70)
**Kids**							
Yes	1308 (64)	3.58 (0.74)	3.69 (0.66)	2.37 (1.07)	4.34 (0.78)	3.76 (1.08)	1.94 (0.59)
No	724 (36)	3.43 (0.82)	3.69 (0.72)	2.52 (1.12)	4.19 (0.88)	3.73 (1.18)	1.92 (0.64)
**Sector**							
Public sector	1663 (82)	3.54 (0.76)	3.65 (0.67)	2.40 (1.09)	4.29 (0.82)	3.71 (1.12)	1.94 (0.62)
Private sector	369 (18)	3.45 (0.82)	3.85 (0.72)	2.52 (1.08)	4.27 (0.82)	3.94 (1.07)	1.87 (0.57)
**Rank**							
Physicians in training	654 (32)	3.50 (0.77)	3.54 (0.69)	2.29 (1.03)	4.42 (0.76)	3.71 (1.11)	2.02 (0.64)
Specialist physician	823 (41)	3.58 (0.76)	3.73 (0.70)	2.45 (1.09)	4.24 (0.84)	3.82 (1.10)	1.92 (0.60)
Consultant	555 (27)	3.47 (0.80)	3.79 (0.63)	2.54 (1.14)	4.19 (0.83)	3.68 (1.12)	1.85 (0.58)
**Work site**							
Primary care facility	893 (44)	3.64 (0.78)	3.75 (0.70)	2.56 (1.06)	4.28 (0.83)	3.85 (1.09)	1.94 (0.60)
Hospital	1009 (50)	3.46 (0.73)	3.63 (0.64)	2.23 (1.06)	4.31 (0.79)	3.65 (1.11)	1.92 (0.62)
Other¹	130 (6)	3.29 (0.95)	3.66 (0.85)	2.95 (1.21)	4.13 (0.95)	3.78 (1.25)	1.93 (0.66)
**Clinical work experience (years)**							
< 5	196 (10)	3.46 (0.74)	3.55 (0.65)	2.34 (0.96)	4.49 (0.72)	3.67 (1.14)	2.02 (0.67)
5–10	488 (24)	3.52 (0.75)	3.58 (0.68)	2.27 (1.04)	4.41 (0.75)	3.77 (1.06)	1.99 (0.62)
11–15	440 (22)	3.65 (0.73)	3.65 (0.67)	2.32 (1.07)	4.26 (0.80)	3.73 (1.08)	2.00 (0.63)
>15	908 (45)	3.49 (0.81)	3.79 (0.69)	2.57 (1.14)	4.19 (0.87)	3.76 (1.15)	1.85 (0.58)
**Working hours (avg/week)**							
< 30	101 (5)	3.17 (0.80)	3.78 (0.64)	2.75 (1.07)	4.27 (0.80)	3.94 (1.12)	1.85 (0.61)
30–40	654 (32)	3.41 (0.81)	3.75 (0.67)	2.62 (1.10)	4.36 (0.76)	3.87 (1.09)	1.90 (0.60)
41–50	1066 (52)	3.59 (0.74)	3.65 (0.69)	2.31 (1.05)	4.26 (0.82)	3.69 (1.11)	1.94 (0.61)
> 50	211 (10)	3.74 (0.76)	3.62 (0.72)	2.19 (1.13)	4.15 (0.95)	3.55 (1.19)	2.00 (0.64)
**Regular shift work**							
Yes	1369 (67)	3.55 (0.75)	3.67 (0.67)	2.33 (1.07)	4.32 (0.79)	3.75 (1.10)	1.94 (0.62)
No	663 (33)	3.47 (0.83)	3.72 (0.71)	2.61 (1.11)	4.20 (0.87)	3.75 (1.15)	1.92 (0.60)
**Total**	**2032 (100)**	**3.53 (0.77)**	**3.69 (0.69)**	**2.42 (1.09)**	**4.28 (0.82)**	**3.75 (1.11)**	**1.93 (0.61)**

¹private health care clinics, outpatient clinics (outside hospital), occupational health care clinics (outside hospital)

### Data analysis

Descriptive characteristics and scores of exposure variables (i.e., J-DCS) and outcome (i.e., BAT) were calculated and presented across all sociodemographic and occupational groups using appropriate measures of central tendency and dispersion. We evaluated the Pearson’s correlation coefficient (i.e., > 0.7 or < -0.7) to identify possible multicollinearity [28] between the exposure and outcome variables and found no strong evidence of multicollinearity (please see Supplementary Material). To investigate group differences regarding high burnout risk, univariate analysis was performed using the Chi-square test for categorical variables (i.e., sex, partner, kids, sector, rank, working experience, work site, working hours, shift work) and the Student’s t-test for the continuous variable age. The relevant background variables that exhibited statistical significance in the univariate analysis were included in the binary multivariable regression models.

Binary logistic regression models were used to examine the associations between J-DCS (continuous exposure variables) and risk of high burnout (categorical outcome variable). In order to select the background variables, we adopted stepwise logistic regression, which included the following steps. Firstly, the crude analysis included each J-DCS measure in a separate single-variable logistic regression model. Secondly, each model was adjusted for relevant background variables that exhibited significance in the univariate analysis. Thirdly, all J-DCS measures were included into one adjusted multivariable logistic regression model. Lastly, interaction analysis was performed to investigate a potential moderation of the association between job demands and high burnout risk. This was done by introducing interaction terms (not z-standardized) between job demands and each of the job control and support variables (i.e., one at a time) to the adjusted multivariable model (i.e., job demands x workplace control, job demands x peer support, etc.). Similarly, a three-way interaction term between job demands and the significant job control and support variables (i.e., job demands x workplace control x peer support) was added separately to investigate a possible three-way moderation of the association between job demands and high burnout risk. Results from the regression models were presented as odds ratios (OR) and 95% confidence intervals (CI), and all statistical tests were two-sided, where a p-value of ≤ 0.05 was defined as statistically significant. All analyses were performed in STATA version 17.0 (StataCorp, College Station, TX, USA) [29].

## Results

The study population of the present study consisted of 56% females and a mean age of 46 years (Table 2).

**Table 2:** Prevalence of high burnout risk (BAT-score *≥ 3.02)* acrosss Sociodemographic and Occupational groups of physicians in Sweden

Burnout Assessment Tool (BAT) scores					
	No Burnout Risk		High Burnout Risk		
	*BAT-score < 3.02*		*BAT-score ≥ 3.02*		P-value*
	*n (%)*		*n (%)*		
**Sex**					0.027
Male	857 (96)		36 (4)		
Female	1068 (94)		71 (6)		
**Age (mean (SD))**	46.1 (0.25)		42.4 (0.90)		0.0007
**Partner**					0.001
Yes	1757 (95)		87 (5)		
No	168 (89)		20 (11)		
**Kids**					0.102
Yes	1247 (95)		61 (5)		
No	678 (94)		46 (6)		
**Sector**					0.030
Public sector	1567 (94)		96 (6)		
Private sector	358 (97)		11 (3)		
**Rank**					0.021
Physicians in training	607 (93)		47 (7)		
Specialist physicians	784 (95)		39 (5)		
Consultants	534 (96)		21 (4)		
**Worksite**					0.682
Primary care facility	847 (95)		46 (5)		
Hospital	957 (95)		52 (5)		
Other¹	121 (93)		9 (7)		
**Clinical work experience (years)**					0.012
< 5	177 (90)		19 (10)		
5–10	461 (95)		27 (5)		
11–15	415 (94)		25 (6)		
>15	872 (96)		36 (4)		
**Working hours (avg/week)**					0.907
< 30	96 (95)		5 (5)		
30–40	622 (95)		32 (5)		
41–50	1009 (95)		57 (5)		
> 50	198 (94)		13 (6)		
**Regular shift work**					0.537
Yes	1294 (95)		75 (5)		
No	631 (95)		32 (5)		
**Total**	**1925 (95)**		**107 (5)**		

¹ private health care clinics, outpatient clinics (outside hospital), occupational health care clinics (outside hospital)

* Group comparisons regarding high burnout risk using Chi-square test for categorical variables and Student’s t-test for continuous variable (i.e., age)

### Job demand-control-support and risk of high burnout across background variables

#### Job Demand, Control, and Support

Across sociodemographic groups, female physicians reported higher job demands (mean 3.63, SD 0.74) compared to their male counterparts (mean 3.39, SD 0.80), whereas males reported higher job control (i.e., both workplace and task-level control) as well as support (i.e., both peer- and manager support) (Table 2). Across age categories, physicians in the upper age quartile (i.e., 58–69 years) reported less job demands and higher job control than younger physicians.

Across occupational groups, physicians working in the public sector experienced higher job demands, less job control, and less manager support than physicians within the private sector (Table 2). Hospital-based physicians reported less job demands but also less job control and less manager support than physicians working in primary care. In general, lower demands and higher job control were observed among senior-rank physicians (consultants) and among physicians with more years of clinical work experience (i.e., > 15 years). On the contrary, higher job demands and lower job control were observed among those reporting long working hours (i.e., > 40 h/week) and regular shift work.

#### Burnout

In this study, a total of 5% of physicians were considered at high risk of burnout, differing slightly across sex (i.e., female = 6%, male = 4%) (in). Across occupational groups, 6% of physicians in the public sector and 3% of private sector physicians were at high risk of burnout. Hospital-based physicians and primary care physicians had equal frequencies of high burnout risk (5%). In general, high burnout risk were reported more frequently among more junior rank physicians (i.e., physicians in training and specialists) and physicians with < 15 years of clinical work experience. The frequencies of high burnout risk were similar across working hours and equal across shift work conditions.

Statistically significant group differences regarding high burnout risk were found for age, sex, having a partner, years of clinical working experience, sector and rank (Table 1). These background variables were included in further analyses below.

### Associations between job demand-control-support and burnout among physicians

In the crude analysis, statistically significant associations were found between each of the J-DCS variables and high burnout risk (Model 1, Table 3). Specifically, job demands were associated with increased odds of high burnout risk (OR 3.66, 95% CI 2.67–5.03). Contrary, reduced odds of high burnout risk were found for workplace control and task-level control (OR 0.27, 95% CI 0.20–0.35 and OR 0.61, 95% CI 0.50–0.76, respectively), and peer support and manager support (OR 0.50, 95% CI 0.43–0.59 and OR 0.45, 95% CI 0.37–0.55, respectively). These associations remained at similar statistically significant levels after adding relevant background variables (i.e., age, sex, partner, working experience, sector and rank) to each regression model (Model 2, Table 3).

**Table 3:** Associations between job demand-control-support and high Burnout risk among physicians in Sweden

	J-DCS variables separate		J-DCS variables combined			
	**Model 1 (crude)¹**	**Model 2²**	**Model 3³**	**Model 4⁴**	**Model 5⁵**	**Model 6⁶**
	*OR (95% CI)*	*OR (95% CI)*	*OR (95% CI)*	*OR (95% CI)*	*OR (95% CI)*	*OR (95% CI)*
**Demands**	3.66 (2.67–5.03)**	3.77 (2.71–5.24)**	2.71 (1.91–3.84)**	2.11 (0.40-11.13)	2.38 (0.69–8.17)	2.16 (1.07–4.35)*
**Workplace Control**	0.27 (0.20–0.35)**	0.28 (0.21–0.37)**	0.50 (0.35–0.71)**	0.36 (0.05–2.92)	0.50 (0.35–0.71)**	0.38 (0.17–0.86)*
**Task-level control**	0.61 (0.50–0.76)**	0.65 (0.53–0.81)**	0.95 (0.76–1.20)	0.95 (0.76–1.20)	0.95 (0.76–1.20)	0.95 (0.76–1.20)
**Manager support**	0.50 (0.43–0.59)**	0.51 (0.44–0.60)**	0.83 (0.67–1.03)	0.83 (0.67–1.03)	0.83 (0.67–1.03)	0.82 (0.67–1.02)
**Peer support**	0.45 (0.37–0.55)**	0.42 (0.34–0.51)**	0.61 (0.48–0.77)**	0.61 (0.48–0.77)**	0.53 (0.14–1.96)	0.48 (0.24–0.94)*
**Demands x Workplace Control**				1.08 (0.65–1.79)		
**Demands x Peer support**					1.04 (0.75–1.42)	
**Demands x Peer support x Workplace control**						1.02 (0.97–1.07)

¹ Model 1: each J-DCS variable included in a separate logistic regression model (crude)

² Model 2: each logistic regression in Model 1 adjusted for relevant background variables (i.e., sex, age, partner, sector, rank and working experience)

³ Model 3: all J-DCS variables included in a multivariable logistic regression model, adjusted for relevant background variables (i.e., sex, age, partner, sector, rank and working experience)

⁴ Model 4: interaction term between Demands and Workplace Control added separately to Model 3

⁵ Model 5: interaction term between Demands and Peer Support added separately to Model 3

⁶ Model 6: three-way interaction term between Demands, Workplace Control and Peer Support added separately to Model 3

Bold OR indicates statistical significance (* p-value < 0.05, ** p-value < 0.001)

In Model 3 (Table 3), by forcing all J-DCS variables into one multivariable logistic regression model (adjusted for the relevant background variables), the OR for the association between job demands and high burnout risk decreased yet remained significantly elevated (OR 2.71, 95% CI 1.91–3.84). The OR for workplace control and peer support increased (OR 0.50, 95% CI 0.35–0.71 and OR 0.61, 95% CI 0.48–0.77, respectively). Hence, including demands-control-support simultaneously in one regression model reduced the odds-increasing association between job demands and high burnout risk slightly, while the beneficial associations between workplace control, peer support and high burnout risk decreased. Notably, the CI for the association between job demands and high burnout risk largely overlapped in Models 2 and 3, indicating no buffering impact of workplace control or peer support. Moreover, the associations between manager support, task-level control, and high burnout risk became non-significant. In the final step, interaction terms were added separately (i.e., one by one) into the adjusted multivariable logistic regression model, although no significant interaction effect was identified between either workplace control and job demands (Model 4, Table 3), peer support and job demands (Model 5, Table 3), or in the three-way interaction between workplace control, peer support and job demands (Model 6, Table 3). This indicated that the odds-increasing association between job demands and high burnout risk was not moderated by peer support or workplace control. Additionally, no significant interaction terms were found between job demands and task-level control (OR 1.20, 95% CI 0.84–1.71), and between job demands and manager support (OR 1.02, 95% CI 0.78–1.36), respectively (not presented in Table 3).

## Discussion

This study aimed to explore the work-related factors of the J-DCS model across different groups of physicians in Sweden and to investigate the associations between J-DCS factors and high burnout risk. Additionally, we investigated the potential buffering impact of job control and support on high burnout risk. We identified variations in scores of J-DCS measures and high burnout risk across different sociodemographic and occupational groups of physicians. We found that job demands were significantly associated with increased odds of high burnout risk, whereas reduced odds of high burnout risk were found for workplace control and peer support. However, the interaction analysis revealed no significant moderation by either workplace control or peer support on the association between job demands and high burnout risk, and additionally, no buffering impact was found. Hence, in contrast to the J-DCS theoretical framework, our findings suggest that job demands may have an important risk-increasing association with burnout, irrespective of the level of peer support and workplace control among physicians in Sweden. These findings could be of value for practice- and policymakers in promoting sustainable work environments within the Swedish healthcare sector. To verify these associations and establish causality of antecedents to high burnout risk however, longitudinal studies are required.

The associations between J-DCS factors and high burnout risk identified in the present study align with previous studies in other countries. For example, among Belgian and Lithuanian hospital physicians, job demands were associated with approximately 3- and 5-times increased risk of burnout, respectively, whereas job control and social support at the workplace had inverse relationships with burnout risk [30, 31]. Regarding support, we found that peer support (but not managerial support) had a significant odds-reducing association with high burnout risk. According to previous studies, support at the workplace has the potential to mitigate the negative impact of work-stress exposure among physicians [10–13, 32]. Identifying factors that act protectively to work-stress exposure is crucial to ultimately design preventive measures. Therefore, future research should further investigate the potential beneficial aspects of various types of support (i.e., managerial and peer support) regarding burnout risk among physicians in Sweden.

In line with the J-DCS conceptualization, increasing the supportive elements of the work setting has been underlined as a more feasible stress-mitigating measure than reducing demands [33], since job demands are situational and attributed to the nature of the healthcare work setting [1, 2, 4]. However, in the present study, neither peer support nor workplace control buffered or moderated the association between job demands and high burnout risk, which aligns with recent findings showing a diminished positive impact of job control and support in the presence of high job demands [19]. This emphasizes revising the applicability of the J-DCS model among physicians in Sweden. However, to do that and to establish causality, additional studies are needed to further investigate and confirm the associations between J-DCS and high burnout risk among physicians in Sweden.

High burnout risk has been reported in previous studies on physicians in other countries [1–3, 5, 6]. However, direct comparisons to our results are complicated by a vast methodological heterogeneity in previous studies [6, 34], where the utilization and interpretation of burnout measurement tools have differed largely [16]. Specifically, a systematic review of burnout among physicians by Rotenstein et al. identified 142 unique variations of criteria for the presence of burnout in the included studies [2]. For example, previous studies utilizing the Maslach Burnout Index (MBI) [35] have focused largely on measurements of exhaustion only (i.e., one symptom of burnout) [16]. Restricting the measurement to only one of several symptoms of burnout has received criticism for causing substantial variation in burnout prevalence estimates, limiting the ability to conduct meta-analyses [34]. Contrary, when all three sub-scales of the MBI are used to measure burnout, the prevalence is estimated at 7.7% among European physicians [36], which aligns with the findings of the present study. However, we advocate for the use of BAT to avoid limitations caused by the arbitrary and heterogenous application of the MBI [24, 34].

Notably, in this study, high burnout risk were reported more frequently among early career physicians (i.e., in younger age categories, among physicians in training and those with < 5 years of clinical work experience), which aligns with previous findings [1, 37]. It is important to find ways of mitigating the development of burnout among early career physicians (i.e., physicians in training) [37]. The potential beneficial impact of job control and support may differ across career status and age of physicians. For example, certain elements of the job control aspect (e.g., clinical decision-making ability, autonomy, etc.) increase gradually with working experience [38]. Therefore, future studies should further investigate the potential positive impact of job control and support across different groups of physicians in Sweden.

### Strengths & limitations

A major strength of this study was the large representative sample of physicians in Sweden based on the LOHHCS study cohort. An additional strength was the use of BAT, which allowed for calculations on a global burnout score with a pooled cut-off value for high burnout risk that has been validated in several countries in close proximity to Sweden (e.g., Finland, Netherlands, Belgium) [25]. Hence, limitations of the widely used Maslach burnout inventory tool were avoided, e.g., the restriction to measurements of each dimension of burnout individually [24, 39].

However, several limitations of the present study should be addressed. First, due to questionnaire length restrictions, a validated tool to assess J-DCS specifically (e.g., the 22-item Job Content Questionnaire) [40] was not utilized, complicating the comparability of J-DCS elements between studies. However, core items from the COPSOQ-III were used. The COPSOQ is widely used to study J-DCS factors [41]. Regarding the control dimension of J-DCS, we combined core items from the COPSOQ with additional questions capturing healthcare-specific control. An EFA was utilized to construct feasible variables for the job control aspect. Second, the cross-sectional study design prevents any causal conclusions between exposure to J-DCS and high burnout risk. Third, data was gathered during the COVID-19 pandemic (i.e., the peak of the third wave in Sweden), associated with increased levels of work stress among physicians globally [42]. This may have affected work stress distribution across different occupational groups (e.g., primary care vs. hospital-based physicians). Fourth, data was based on self-report measures, increasing the risk for several biases (e.g., recall bias, common method bias). However, comparisons of self-reported data versus external assessments of job demands and control have shown stability across different levels of psychological distress [43]. Fifth, individual stress-mitigating factors (e.g., individual stress resilience and coping strategies) were not considered, which may affect the individual burnout risk [16, 44]. Last, a majority of the invited physicians declined the survey, although the response rate was sufficient according to power calculations. Therefore, the results may have been affected by attrition bias (e.g., less stressed physicians may be more prone to respond, or vice versa).

## Conclusions

Job demands were associated with high burnout risk among physicians in Sweden. While peer support and workplace control had inverse associations with high burnout risk, no moderation or buffering impact on the association between job demands and high burnout risk was found. Additional studies in longitudinal settings are needed to further investigate and confirm these associations.

## Electronic supplementary material

Below is the link to the electronic supplementary material.

Supplementary Material 1

Supplementary Material 2

## Data Availability

Data used in the present study is available upon reasonable request.
