# Supplementary material for: Associations between job demand-control-support and high burnout risk among physicians in Sweden: a cross-sectional study
Source: J Occup Med Toxicol. 2024 Oct 29;19:42. doi: 10.1186/s12995-024-00441-6 (PMC11520855; doi:10.1186/s12995-024-00441-6)
Supplement: Supplementary file 1 — Supplementary Material 1 [file 12995_2024_441_MOESM1_ESM.docx]

| **Supplementary Table 1:** Pearson's Correlation Coefficients Between the Variables of J-DCS and BAT | | | | | | | | | | |  |
| --- | --- | --- | --- | --- | --- | --- | --- | --- | --- | --- | --- |
|  |  |  |  |  |  |  |  |  |  |  | |
|  | **1** | **2** | **3** | **4** | **5** | **6** |  |  |  |  | |
| **Demands (1)** | 1 |  |  |  |  |  |  |  |  |  | |
| **Workplace Control (2)** | -0.30 | 1 |  |  |  |  |  |  |  |  | |
| **Task-level control (3)** | -0.27 | 0.34 | 1 |  |  |  |  |  |  |  | |
| **Manager support (4)** | -0.27 | 0.54 | 0.29 | 1 |  |  |  |  |  |  | |
| **Peer support (5)** | -0.19 | 0.31 | 0.14 | 0.42 | 1 |  |  |  |  |  | |
| **BAT (mean score) (6)** | 0.45 | -0.44 | -0.26 | -0.37 | -0.30 | 1 |  |  |  |  | |
|  |  |  |  |  |  |  |  |  |  |  | |
| *BAT = Burnout Assessment Tool (i.e., primary outcome)*  *J-DCS = Job Demand-Control-Support (i.e., exposure variables)* | | | | | | |  |  |  |  | |
