# Supplementary material for: Associations between job demand-control-support and high burnout risk among physicians in Sweden: a cross-sectional study
Source: J Occup Med Toxicol. 2024 Oct 29;19:42. doi: 10.1186/s12995-024-00441-6 (PMC11520855; doi:10.1186/s12995-024-00441-6)
Supplement: Supplementary file 2 — Supplementary Material 2 [file 12995_2024_441_MOESM2_ESM.docx]

| **Supplementary Table 2.** Exploratory Factor Analysis (EFA) output with rotated factor loadings of each Job Control item. | | | |
| --- | --- | --- | --- |
|  |  |  |  |
| **Job Control items** | **Item description** | **Factor 1*** | **Factor 2*** |
| *COPSOQ III* |  | *Task-level control********* | *Workplace control********* |
| i) | Workplace information regarding decisions, changes, and plans | 0.1461 | **0.7078** |
| ii) | Workplace facilitation of efficient work | 0.1378 | **0.7407** |
| *Professional autonomy* | |  |  |
| iii) | Workplace facilitation of clinical decision-making | 0.1728 | **0.4434** |
| *Additional items* |  |  |  |
| iv) | Ability to impact the number of patient consultations per day | **0.7795** | 0.1607 |
| v) | Ability to impact the time frame of each consultation | **0.8192** | 0.1615 |
| vi) | Ability to impact the time for administrative work and documentation | **0.7478** | 0.2733 |
|  |  |  |  |
| **Factor loadings ≥ 0.40 (indicated by bold) were retained for each factor structure* | | | |
| *********Job Control variable names corresponding to the items of each factor structure* | | | |
